# Supplementary material for: Short-term hypercaloric carbohydrate loading increases surgical stress resilience by inducing FGF21
Source: Nat Commun. 2024 Feb 5;15:1073. doi: 10.1038/s41467-024-44866-3 (PMC10844297; doi:10.1038/s41467-024-44866-3)
Supplement: Supplementary file 1 — Supplementary Information [file 41467_2024_44866_MOESM1_ESM.pdf]

Supplementary information

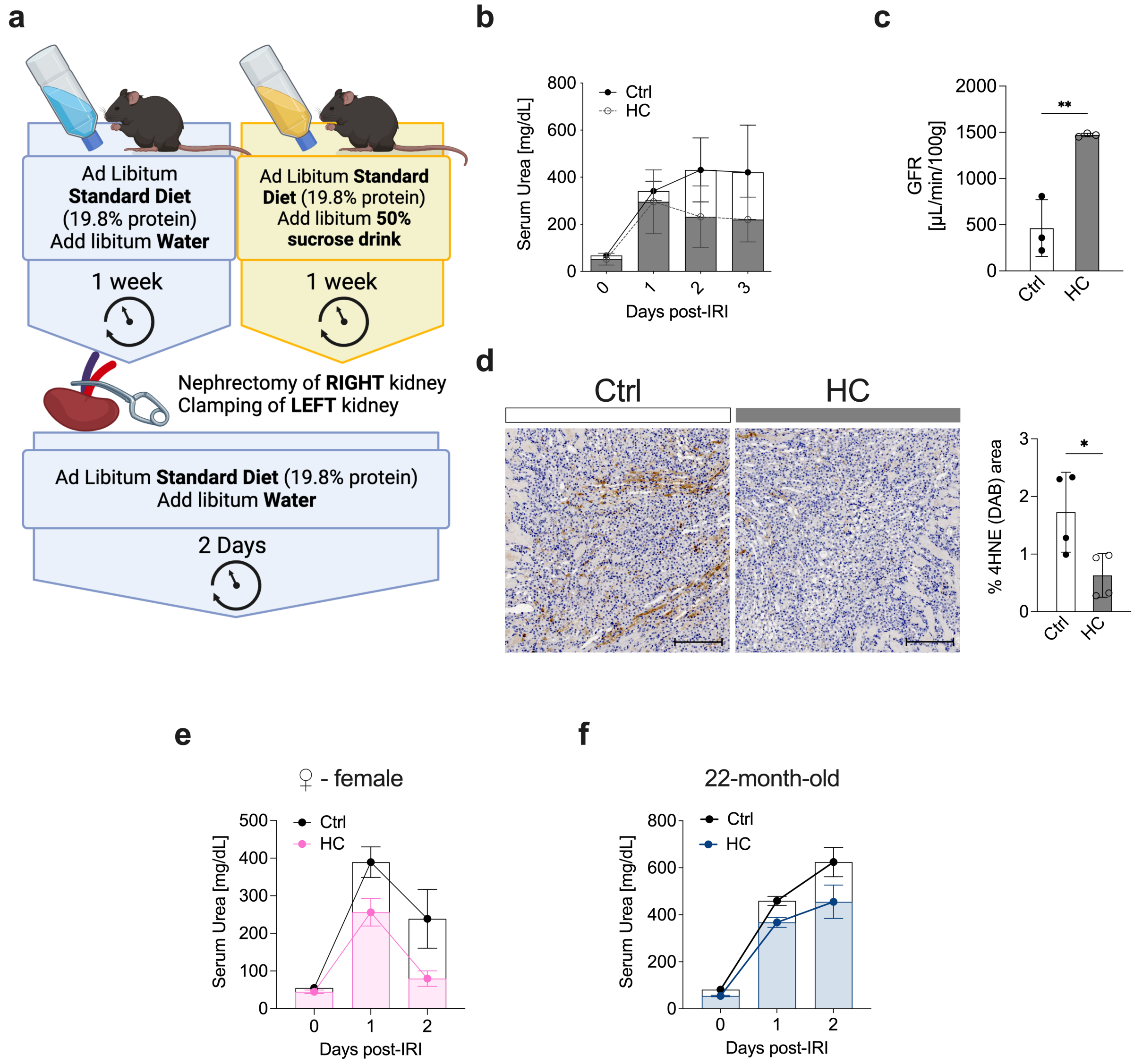

Supplementary Data Fig.1. Additional data for the protective effect induced by the high-sucrose diet.

a. Experimental setup. Ad-libitum fed C57BL/6 mice were given free access to water (Ctrl) or to a 50% sucrose water solution (HC) for one week prior to renal IRI surgery. Created with BioRender.com.

b. Serum urea levels at the indicated time post-renal IRI after one week of preconditioning with control (Ctrl) or 50% sucrose drink (HC).

c. Glomerular filtration rate (GFR) at day 2 post-renal IRI.

d. Representative cross sections of 4HNE-stained kidneys (left; DAB; scale bar 100μm) and percentage area of reactive oxygen species 4HNE staining (right) in kidney at day 2 post-renal IRI.
e. Serum urea levels at the indicated time post-renal IRI after one week of preconditioning with control (Ctrl) or 50% sucrose drink (HC).
f. Serum urea levels at the indicated time post-renal IRI after one week of preconditioning with control (Ctrl) or 50% sucrose drink (HC).

*p values for a. were calculated with unpaired two-tailed T-test, **$p < 0.01$. In b-d, experiments were carried out in 10-weeks old male mice; in e, in 10-weeks old female mice ; in f, in 22-months old male mice. Sample sizes: (a–d), n = 8 for all conditions. Data in all panels are shown as mean ± SD. See also Fig. 1

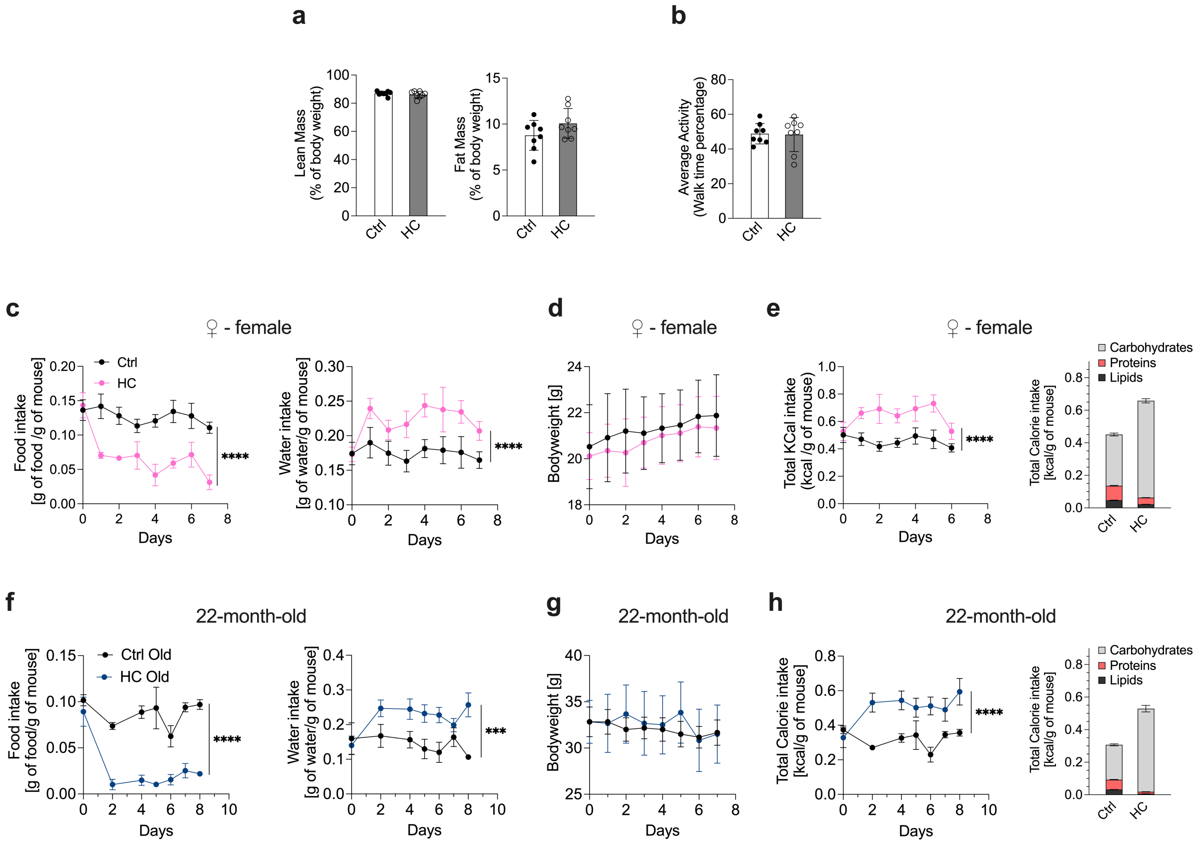

Supplementary Data Fig.2. Additional data for the high-sucrose diet-induced protective effects.

a. Percentage of lean (left) and fat mass (right) after one week of preconditioning with water (Ctrl) or 50% sucrose drink (HC).

b. Average spontaneous activity after one week of preconditioning with water (Ctrl) or 50% sucrose drink (HC).

c. Food (left) and water (right) intake (normalized by body weight) in mice given *ad libitum* access to water (Ctrl) or 50% sucrose drink (HC).

d. Body weight at the indicated time of mice given ad libitum access to indicated diet.

e. Total calorie intake (normalized by body weight) at the indicated time in mice and per macronutrient (right; protein, carbohydrate, fat).

f. Food (left) and water (right) intake (normalized by body weight) of mice given *ad libitum* access to water (Ctrl) or 50% sucrose drink (HC).

g. Body weight at the indicated time of mice given *ad libitum* access to water (Ctrl) or 50% sucrose drink (HC).

h. Total calorie intake (normalized by body weight) at the indicated time of mice and per macronutrient (right; protein, carbohydrate, fat).

*p values for c, e, f, h were calculated with two-way repeated measures (RM) ANOVA with Geisser-Greenhouse correction, ***$p < 0.001$ ****$p < 0.0001$. In a-b, experiments were carried out in 10-weeks old male mice; in c-e, in 10-weeks old female mice ; in f-h, in 22-months old male mice. Sample sizes: (a), n = 6 -7 for 10-week-old female mice; (b), n = 6-7 for 22-month-old male mice; (c–e), n = 6-7 for 10-week-old; (f–h), n = 8 for 22-month-old male mice in all conditions. Data in all panels are shown as mean ± SD. See also Fig. 2 and 2 and Supplementary Data Fig.3.

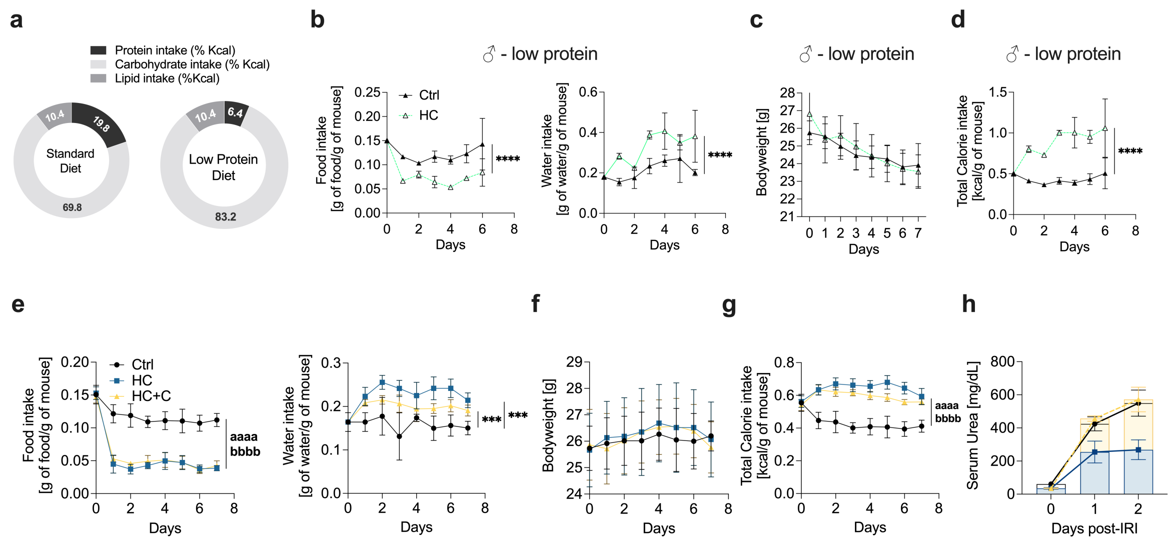

Supplementary Data Fig. 3. Additional data for the high-sucrose diet-induced metabolic effects of the low protein diet.

a. Ratio of each macronutrient in the preconditioning control diet and low protein diet.

b. Food (left) and water (right) intake (normalized by body weight) in mice given *ad libitum* access to low protein diet (LP) with water (Ctrl) or 50% sucrose drink (HC).

c. Body weight in mice given *ad libitum* access to the indicated low protein diet.

d. Total calorie intake (normalized by body weight) at the indicated time given *ad libitum* access to the indicated low protein diet.

e. Food (left) and water (right) intake (normalized by body weight) in mice given *ad libitum* access to control (Ctrl), 50% sucrose drink (HC) or 50% sucrose drink with cysteine oral gavage (HC + cysteine).

f. Body weight in mice given *ad libitum* access to the indicated diet.

g. Total calorie intake (normalized by body weight) at the indicated time in mice given *ad libitum* access to the indicated low protein diet.

h. Serum urea levels at the indicated time post-renal IRI in mice preconditioned for 1 week with the indicated diet.

*p values of b, d, e, g were calculated with two-way RM ANOVA with Geisser-Greenhouse correction, ***p < 0.001 ****p < 0.0001 aaaa p < 0.0001 HC vs. Ctrl bbbb p < 0.0001 HC+C vs. Ctrl. In all panels, experiments were carried out in 10-week old male

mice. Sample sizes: (b–d), n = 6 in all conditions; (e–h), n = 8 in all conditions. Data in all panels are shown as mean ± SD. See also Fig.2 and Supplementary Data Fig.2.

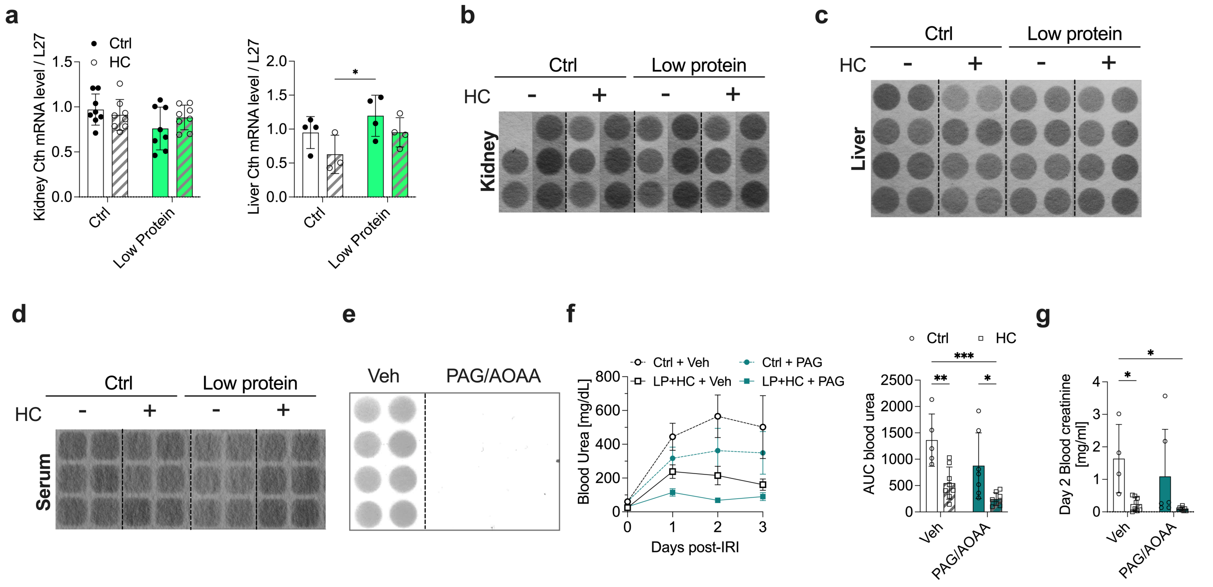

Supplementary Data Fig.4. Additional data for the high-sucrose diet-induced protective effects.

a. Relative Cth (cystathionine gamma-lyase) mRNA levels after one week of preconditioning with control or low protein diet (LP) with control (Ctrl) or 50% sucrose drink (HC).
b. Representative images of lead acetate assay in the kidney, (c) liver and (d) serum after one week of preconditioning on the indicated diet.
e. Representative images of lead acetate assay in the liver of mice treated with Vehicle (Veh) or the H_2S inhibitor propargylglycine (PAG) + amino-oxyacetic acid (AOAA) for one week.
f. Serum urea levels (left) and AUC (right) at the indicated time post-renal IRI after one week of preconditioning with the indicated diet and treatment.
g. Serum creatinine levels at day 2 post-renal IRI after one week of preconditioning with the indicated diet and treatment.

*p values of a,f,g were calculated with two-way ANOVA followed by a Tukey's post hoc analysis, *$p < 0.05$ **$p < 0.01$ ***$p < 0.001$. In all panels, experiments were carried out in 10-week old male mice. Sample sizes: (a), n=8 for the kidney and n= 4 for the liver. (b), n=3 for all conditions with 2 technical replicates; (c), n=4 for all conditions with 2 technical

replicates; (d), n=3 for all conditions with 2 technical replicates; (c), n=4 for all conditions with 2 technical replicates; (f–g), n=8 for all conditions. Data in all panels are shown as mean ± SD. See also Fig. 3 and3 and Supplementary Data Fig.5.

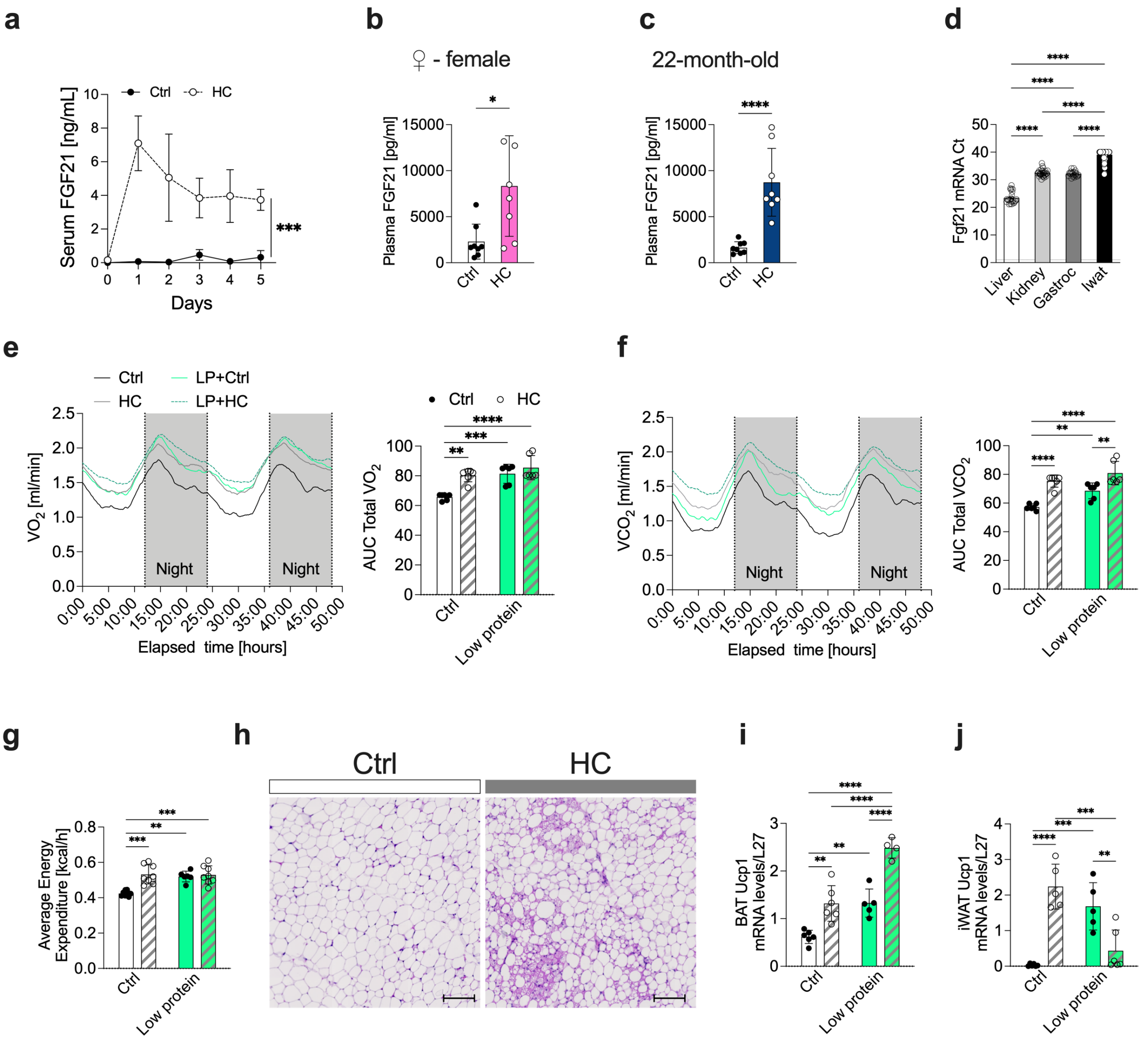

Supplementary Data Fig.5. Metabolic and adipose tissue-related effects of the high-sucrose diet.

a. Serum FGF21 levels over time of mice given *ad libitum* access to water (Ctrl) or 50% sucrose drink (HC).

b. Serum FGF21 levels after one week of preconditioning with control (Ctrl) or 50% sucrose water (HC).

c. Serum FGF21 after one week of preconditioning with control (Ctrl) or 50% sucrose water (HC).

d. FGF21 mRNA levels (cycle threshold; Ct) in the indicated tissue after one week of preconditioning with 50% sucrose drink (HC).

e. VO_2 consumption over time (left), VO_2 (AUC; right) and (f) VCO_2 consumption over time (left), VCO_2 (AUC; right) after 6-7 days of preconditioning with control or low protein diet (LP) with control (Ctrl) or 50% sucrose drink (HC). Each light/dark bar represents a 12-hour duration.

g. Energy expenditure after one week of preconditioning with the indicated diet.

h. Representative sections of H&E-stained inguinal white adipose tissue (iWAT; scale bar 100µm) after one week of preconditioning with the indicated diet.

i. Relative Ucp1 mRNA levels in the brown adipose tissue (BAT) and (j) in the iWAT after one week of preconditioning with the indicated diet.

*p values of a-c were calculated with one-way ANOVA, d with two-way RM ANOVA with Geisser-Greenhouse correction and e-j with two-way ANOVA followed by a Tukey's post hoc analysis, **p < 0.01 ***p < 0.001 ****p < 0.0001. In a and d-i, experiments were carried out in 10-weeks old male mice; in b, in 10-weeks old female mice ; in c, in 22-months old male mice. Sample sizes: (a-b), n=8, (c); n=32 for the kidney, n= 24 for the liver, n=22 for the gastrocnemius, n=48 for the iWAT; (d), n=4 for all conditions; (e–j), n=6 for all conditions. Data in all panels are shown as mean ± SD. See also Fig.3 and Supplementary Data Fig.4

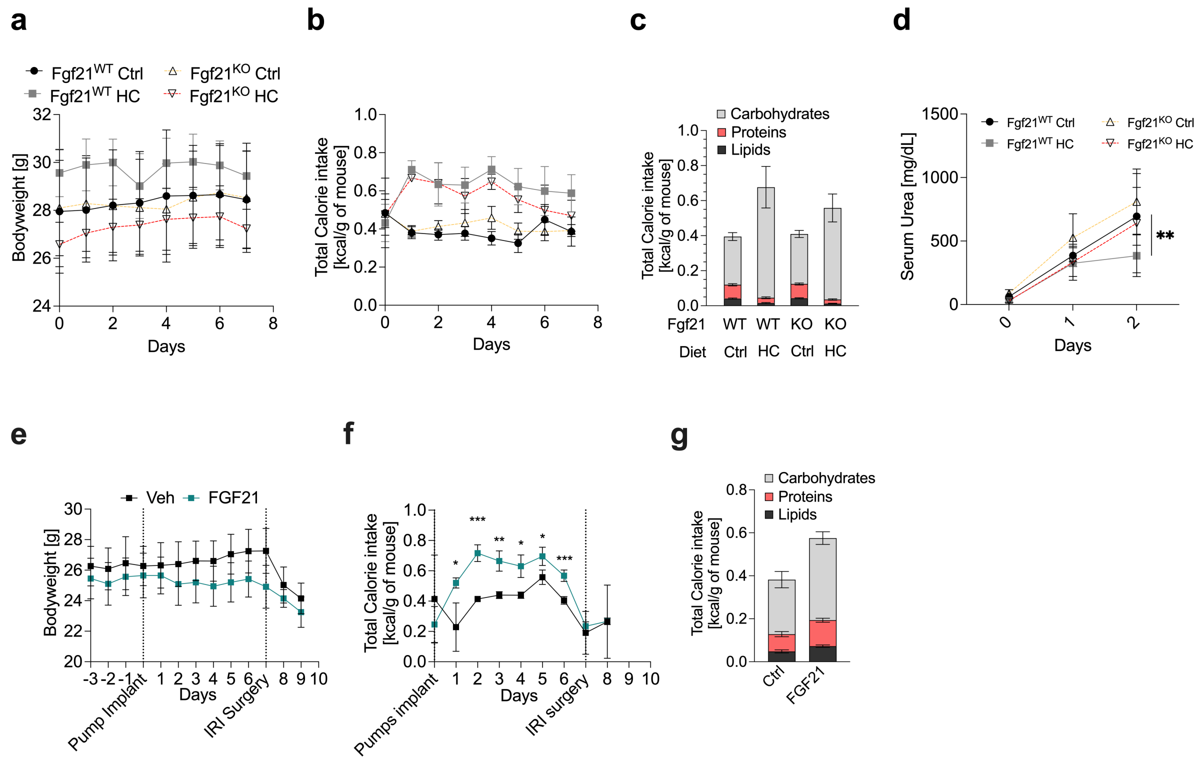

Supplementary Data Fig.6. Metabolic effects of carbohydrate loading in FGF21^{KO} mice.

a. Body weight of Fgf21^{WT} and Fgf21^{KO} mice given *ad libitum* access to to water (Ctrl) or 50% sucrose drink (HC).

b. Total calorie intake (normalized by body weight) at the indicated time and (c) per macronutrients (protein, carbohydrate, fat) of Fgf21^{WT} and Fgf21^{KO} mice preconditioned with the indicated diet.

d. Serum urea levels at the indicated time post-renal IRI of Fgf21^{WT} and Fgf21^{KO} mice preconditioned for one week with the indicated diet.

e. Body weight of mice given *ad libitum* access to a control diet (Ctrl) and implanted with osmotic pumps containing either NaCl (Ctrl) or human recombinant FGF21 (FGF21; 1mg/kg/day) for 7 days prior to renal IRI surgery.

f. Total calorie intake (normalized by body weight) at the indicated time and (g) per macronutrients (protein, carbohydrate, fat) of mice treated for one week with the indicated treatment.

*p values for d. were calculated with two-way RM ANOVA with Geisser-Greenhouse correction and f. with two-way ANOVA followed by a Sidak's post hoc analysis, *$p < 0.05$ **$p < 0.01$ ***$p < 0.001$. In all panels, experiments were carried out in 10-weeks old male mice. Sample sizes: (a-d), n = 8-22, n=18 for $FGF21^{WT}$ Ctrl, n= 12 for $FGF21^{WT}$ HC, n= 22 $FGF21^{KO}$ Ctrl, n= 8 for $FGF21^{KO}$ HC; (e–g), n= 6-8, n= 8 Ctrl and n= 6 FGF21 treated group. Data in all panels are shown as mean ± SD. See also Fig.4 and 5.

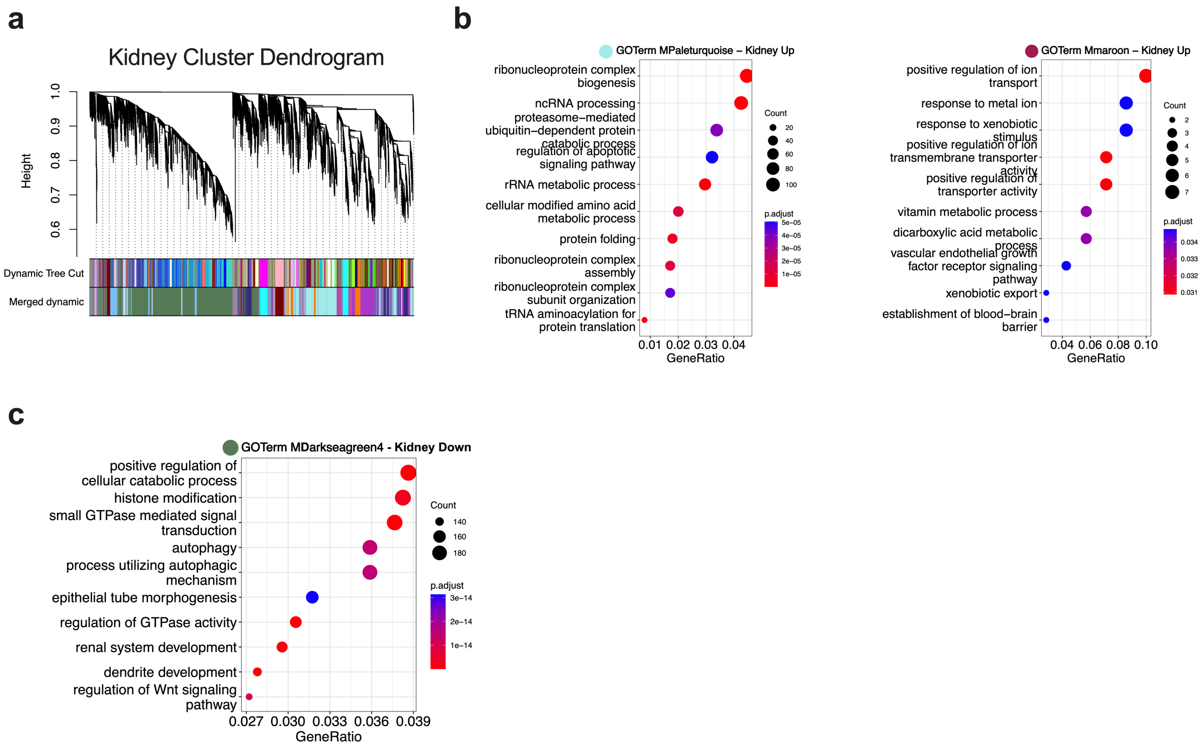

Supplementary Data Fig.7.

a. Dendrogram of kidney clusters displaying gene expression similarities among samples of 10-week-old male mice. Merge cluster dynamic tree cut algorithm identifies distinct color-coded modules at the bottom.

b. Gene Set Enrichment Analysis (GSEA) of significantly positively correlated module MPaleturquoise and MMaroon color with serum FGF21. Size of dots corresponds to the number of genes in the pathway, while color represents the significance level, with red indicating a low significance and blue a high significance.

c. Dotplot of GSEA of modules with negative correlation with serum FGF21 in Darkseagreen4 color.

See also Fig. 6 and Supplementary Data Fig.8.

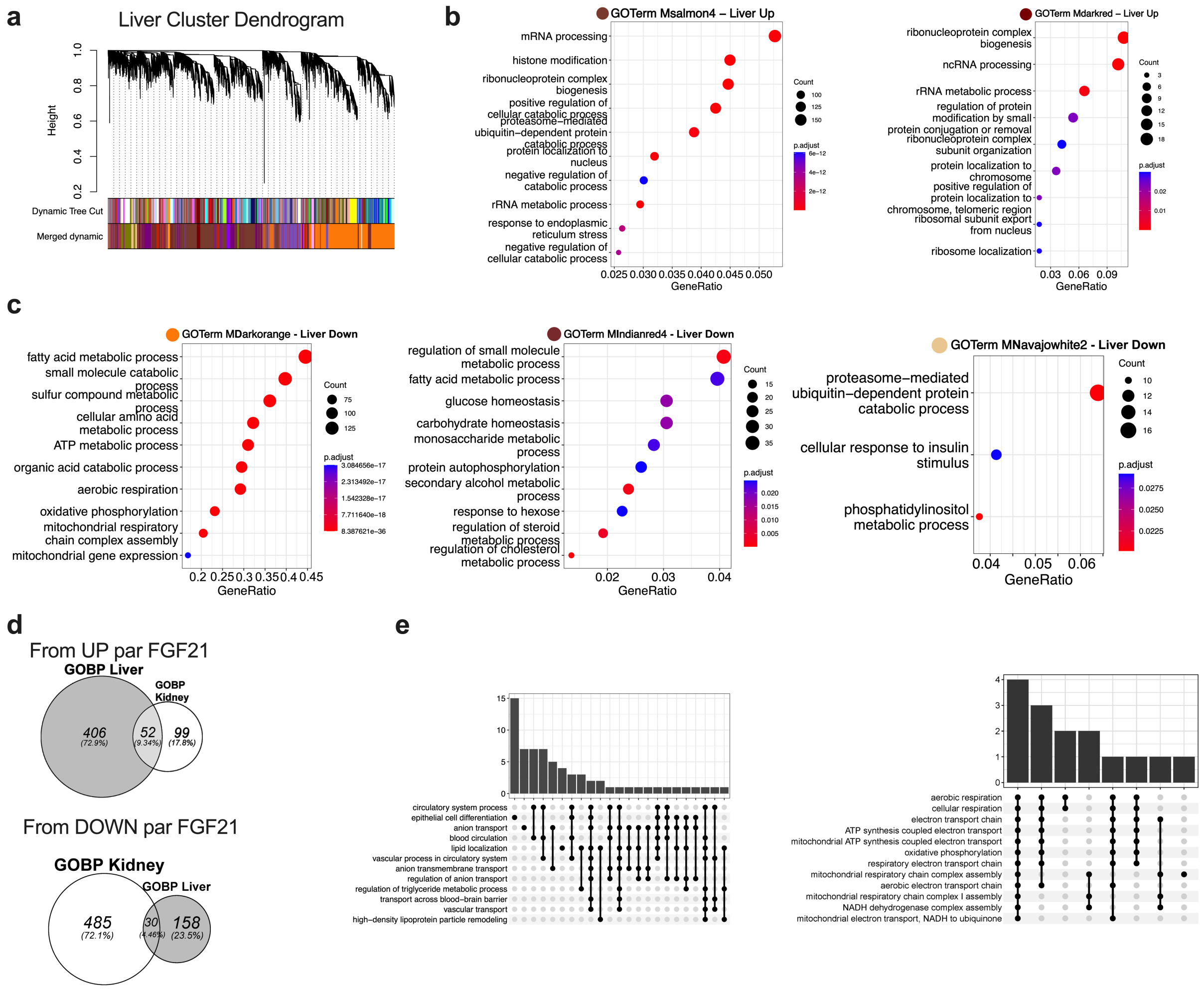

Supplementary Data Fig.8.

a. Dendrogram of liver Cluster displaying gene expression similarity among samples. Merge cluster dynamic tree cut algorithm identifies distinct color-coded modules at the bottom.

b. Dotplot of GSEA of modules with positive correlation with serum FGF21 in Salmon4 and Darkred color.

c. Dotplot of GSEA of modules with negative correlation with serum FGF21 in Darkorange, Indianred4 and Navajowhite2 color.

d. Venn diagram of up-regulated (top) and down-regulated (bottom) correlated modules with serum FGF21 between liver and kidney. The diagram shows the number of shared and unique modules across the two tissues.

e. Upset plot of significant genes positively (left) and negatively (right) correlated with protein dilution intake, identified from the slope > 9 and r2 > 0.7. The plot displays the distribution of the significant GOBP term in the different intersecting sets.

See also Fig.6 and Supplementary Data Fig.7

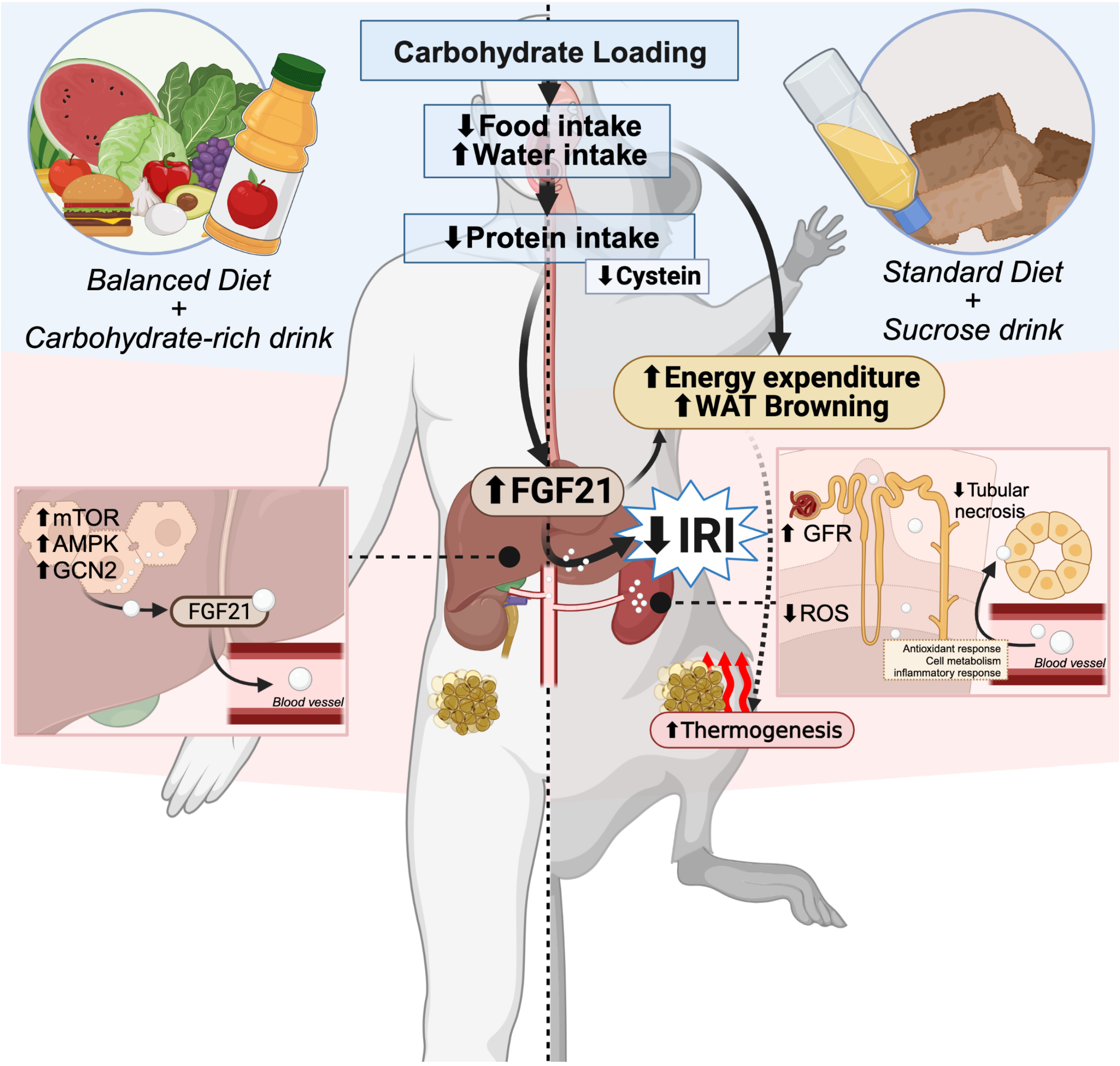

Supplementary Data Fig.9. Proposed Model. Created with BioRender.com.

Target	Specie	Forward Sequence 5'-3'	Reverse Sequence 5'-3'	product size
Krt20	mus musculus	ACAGTTCGAGAGACAGAGTCAA	CTGAGGTGGGACTGCAACTC	138
Rpl27a	mus musculus	TGATGTTGTTCGATCAGGCT	ATTTGGCCTTCACGATGACA	80
Cth	mus musculus	TCCCTTCATCATGCTGAGACC	GTCATGATTGCCGGAAGCTC	104
Fgf21	mus musculus	CACCGCAGTCCAGAAAGTCT	AGAGCTCCATCTGGCTGTTG	110
Cyp2b9	mus musculus	CTGCCCTTCTCCACAGGAAA	CTTTGGAGCAACAGGGCTTG	120
Fmo3	mus musculus	GCCATGTAGCTCAGAAGGTCA	AGATGGCGGTGGGTAAGTTG	144
Mt1	mus musculus	CTCCGTAGCTCCAGCTTCAC	AGGAGCAGCAGCTCTTCTTG	137
Nt5e	mus musculus	GCAGCATTCCTGAAGATGCG	CTCCCGAGTTCCTGGGTAGA	88
Cyp2b10	mus musculus	GTACACAGACCGTCAGTTCTT	AGAGAAGAGCTCAAACATCTGG	97
Cyp17a1	mus musculus	GGAGAGTTTGCCATCCCGAA	TCTAAGAAGCGCTCAGGCAT	113
Igfbp1	mus musculus	TTTATCACAGCAAACAGTGTGAGA	CATGGGTAGACACACCAGCAG	70
Vldlr	mus musculus	GGCTGGATTCCAAGTTGCAC	TCCAGTAGACGCGATCCTCA	132
Lpl	mus musculus	GCGTAGTTCCAGCAGCAAAG	AGAAATCTCTTCCCGCGTCTG	142
Tmem176a	mus musculus	TGCCTCCAGGGACAGACGAT	AGTTTGGCCAGAGCAGACTC	125
Hamp2	mus musculus	AGGGCAGACATTGCGATCC	ACAGCACTGACAGCAGAATC	84
Ucp1	mus musculus	CGGGCATTCAGAGGCAAATC	CCGAGAGAGGCAGGTGTTTC	103
Hmgb1	mus musculus	ACCTATATCCCTCCCAAAGGG	GAGGAAGAAGGCCGAAGGAG	84
Slc22a26	mus musculus	CTTATACCCACAAGGTGGCTGA	ACAACCTCCATGGTTAGGACAT	143

Supplementary Data Table 1. Primers for qPCR.
